# COVID‐19: lambda interferon against viral load and hyperinflammation

**DOI:** 10.15252/emmm.202012465

**Published:** 2020-05-25

**Authors:** Evangelos Andreakos, Sotirios Tsiodras

**Affiliations:** ^1^ Laboratory of Immunobiology Center for Clinical Experimental Surgery and Translational Research Biomedical Research Foundation of the Academy of Athens Athens Greece; ^2^ Airway Disease Infection Section National Heart and Lung Institute Imperial College London London UK; ^3^ 4^th^ Department of Internal Medicine Attikon University Hospital University of Athens Medical School Athens Greece; ^4^ Hellenic Centre for Disease Control and Prevention Athens Greece

**Keywords:** COVID‐19, interferon, viral infection, hyperinflammation, cytokine storm, Immunology, Microbiology, Virology & Host Pathogen Interaction, Respiratory System

## Abstract

Coronavirus disease 2019 (COVID‐19), triggered by the betacoronavirus SARS‐CoV‐2, has become one of the worst pandemics of our time that has already caused more than 250,000 deaths (JHU data‐05/06/2020, https://coronavirus.jhu.edu/). Effective therapeutic approaches are urgently needed to reduce the spread of the virus and its death toll. Here, we assess the possibility of using interferon‐lambda (IFNλ), a third type of interferon sharing low homology with type I IFNs and IL‐10, for treating COVID‐19 patients. We discuss the unique role of IFNλ in fine‐tuning antiviral immunity in the respiratory tract to achieve optimal protection and minimal host damage and review early evidence that SARS‐CoV‐2 may impair IFNλ induction, leading to a delayed type I IFN‐dominated response that triggers hyperinflammation and severe disease. We also consider the potential windows of opportunity for therapeutic intervention with IFNλ and potential safety considerations. We conclude that IFNλ constitutes a promising therapeutic agent for reducing viral presence and hyperinflammation in a single shot to prevent the devastating consequences of COVID‐19 such as pneumonia and acute respiratory distress syndrome (ARDS).

## Graphical abstract

Can we treat COVID‐19 with IFNλ? E. Andreakos and S. Tsiodras discuss how SARS‐CoV‐2 may impair IFNλ induction, leading to a delayed type I IFN‐dominated response that triggers hyperinflammation and severe disease.

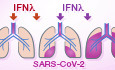

SARS‐CoV‐2 first appeared in December 2019 in Wuhan, Hubei, China, when a number of people presented with a disease resembling viral pneumonia, now termed COVID‐19. It has rapidly spread to all continents and infected millions of people worldwide. In most cases, COVID‐19 clinically manifests with flu‐like symptoms such as fever, headache, and dry cough, and usually runs its course as a mild or uncomplicated illness, eventually resolving spontaneously (Guan *et al*, 2020). However, 15% of patients develop severe pneumonia that requires hospitalization and oxygen support, and 5% of them need admission to an intensive care unit (ICU). This is the result of ARDS, a type of respiratory failure characterized by a rapid and widespread hyperinflammatory response in the lungs that impairs the gas exchange function and leads to multiorgan failure and death. At that stage, mechanical ventilation is the main treatment option to keep the lung functioning while giving the body time to fight the underlying cause. Still, more than half of the patients may die. Understanding what causes ARDS in COVID‐19 and developing therapeutic options for preventing it from happening or reducing its intensity is key to saving thousands of lives.

## Underlying pathophysiology and challenges to treatment

Virally triggered ARDS is characterized by capillary damage and plasma leakage to the alveolar sacs, which disrupts the blood–air barrier and severely impairs blood oxygenation. This can occur directly as a result of viral damage, or indirectly by overactivation of the immune system that triggers the infiltration of immune cells such as neutrophils and macrophages into the lung along with a “cytokine storm”—the excessive or uncontrolled production of cytokines such as TNF, interleukin (IL)‐1β, IL‐6, IL‐12, and IFNγ, and chemokines such as IL‐8, MCP‐1, and IP‐10. This is, in principle, a protective response to limit virus spread but ends up doing more harm than good. Although some of the details may differ, cytokine storms are a common complication of respiratory infections caused by influenza A, SARS‐CoV, and MERS‐CoV viruses, and SARS‐CoV‐2 is no exception (Zhang *et al*, 2020). We can therefore use knowledge from previously studied severe lung infections to identify potential therapeutic targets and devise novel therapeutic strategies.

Existing biologicals targeting cytokines such as IL‐1β and IL‐6 or even inhibitors of cytokine signaling components such as JAK are promising therapeutics to prevent the hyperinflammatory response. Off‐label treatments as well as controlled trials have been initiated (Zhang *et al*, 2020). Yet, the identification of those patients who would mostly benefit, the most appropriate treatment (e.g. anti‐IL‐1 vs anti‐IL‐6‐targeting agents), and the optimal timing of administration so as to not compromise host defenses are important hurdles that will have to be overcome.

## IFNλs for fine‐tuning the antiviral response and preventing the cytokine storm

With the completion of the human genome project, a third type of interferons termed lambda (IFNλs) was identified. In humans, this comprises four members, IFNλ1/IL‐29, IFNλ2/IL‐28A, IFNλ3/IL‐28B, and IFNλ4, all of which signal through a unique heterodimeric receptor complex consisting of IFNLR1 (IFNLRA, IL‐28RA), and IL10R2 (IL‐10RB) (Andreakos *et al*, 2019). IFNλs share low homology with type I IFNs and IL‐10, and exhibit potent antiviral activity, yet their functional importance in the context of health and disease has been difficult to analyze.

Recently, we demonstrated that IFNλs are critical for maintaining a balanced antiviral response in the respiratory tract. They are induced at lower viral burden before type I IFNs to limit the initial infection by inducing viral resistance to cells and helping them deal with the virus load (Galani *et al*, 2017). IFNλs lack the strong pro‐inflammatory effects of type I IFNs and are rather tissue‐protective and anti‐inflammatory. Indeed, if an infection escapes the IFNλ control, an antiviral pro‐inflammatory response driven by type I IFNs occurs at the expense of immunopathology as seen in animal models lacking a functional IFNλ receptor (Galani *et al*, 2017). Conversely, administration of recombinant or pegylated forms of IFNλ suppresses viral replication while stopping the “cytokine storm” from developing (Davidson *et al*, 2016; Galani *et al*, 2017). Further, IFNλs do not compromise the adaptive immune response, but rather stimulate T helper 1, cytotoxic T cell, and antibody responses that are essential for developing long‐term immunity (Koltsida *et al*, 2011; Ye *et al*, 2019). IFNλs therefore act together with type I IFNs to fine‐tune the antiviral immune response for optimal protection against infection while minimizing collateral damage.

## IFNλs for preventing the cytokine storm in COVID‐19

IFNλs have broader implications for the pathogenicity of respiratory infections such as COVID‐19. If a patient gets exposed to higher viral load or is more sensitive to viral entry—by expressing higher levels of the viral receptor for example—the immune system responds with higher type I IFN levels to induce a stronger defense which in turn can cause tissue damage. Alternatively, some viruses are able to suppress IFNλs over type I IFNs, thereby inducing inflammation. Similarly, if a patient has some genetic predisposition for producing more type I IFNs or less IFNλs or is receiving medication affecting the cytokine balance or the viral clearance pathway, a stronger inflammatory response will develop.

**Figure 1:**
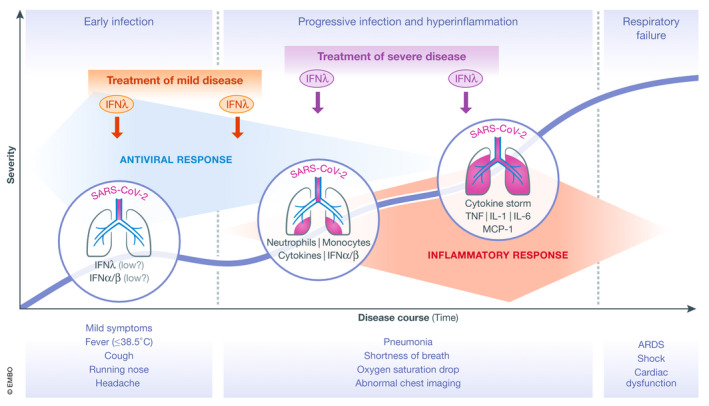
Immunopathogenesis of COVID‐19 and potential windows of opportunity for therapeutic intervention with IFNλ Schematic showing the disease course of COVID‐19 in relation to its clinical and immunopathological characteristics. The thick blue line indicates the progressive incline in disease severity in vulnerable patient populations when untreated. Therapeutic intervention with a single or repeated dose of IFNλ in patients with mild disease (marked with red), or later on in patients with various degrees of pneumonia severity (marked with purple) appears to be the most promising approach. ARDS: Acute Respiratory Distress Syndrome; Mon, monocytes; Neu, neutrophils.

Although the specifics are not known for COVID‐19, SARS‐CoV‐2 can persist in patients for extended periods of times, shedding viral RNA and infectious virus particles for more than 10 days after initial symptoms (Wolfel *et al*, 2020). It is also a fact that MERS‐CoV potently suppresses IFNλ production from human epithelial cells through the expression of non‐structural protein 4a (NS4a), and NS4b with little effect on type I IFNs (Comar *et al*, 2019). As NS4a and NS4b proteins are highly conserved between betacoronaviruses including SARS‐CoV and SARS‐Cov‐2 (Srinivasan *et al*, 2020), there is no reason to suspect that SARS‐CoV‐2 will be different. Moreover, an experimental animal model of SARS‐CoV infection showed that type I IFNs were induced late and triggered a robust inflammatory response with high TNF, IL‐6, and MCP‐1 levels causing dramatic lethality (Channappanavar *et al*, 2016). These pieces together suggest that inefficient viral clearance, possibly owing to impaired production of IFNλs, and secondary induction of type I IFNs driving the “cytokine storm” leading to severe pneumonia and ARDS, is a very plausible scenario (Fig 1). We therefore propose the administration of IFNλs to treat persistent virus presence in COVID‐19 patients while preventing or tampering the “cytokine storm” and its devastating consequences.

## Windows of opportunity for intervention with IFNλs

If IFNλs are to be used for treating COVID‐19 patients, which would be the ideal settings to balance the risks and benefits? Certainly, treatment should be reserved to patients at high risk of developing a severe disease, that is, patients with diabetes, hypertension, obesity, or cardiovascular diseases, and the elderly. There is a good window of opportunity to interfere with the disease process before it is too late as the virus has a much longer incubation time between first disease symptoms and development of pneumonia and/or ARDS. The evaluation of IFNλ treatment should focus on early mild disease in high‐risk patients before any signs of pneumonia, or in patients with developing or established pneumonia with or without oxygen support (Fig 1). Whether treatment can still benefit patients already in the ICU, beyond the first couple of days, is more difficult to say, because at this stage the damage to the alveolar epithelial–endothelial barrier has largely taken place, the viral load has dropped and the “cytokine storm” is declining. Combination treatment is another option to be considered. The National Health Commission of the People's Republic of China guidelines for the treatment of COVID‐19 proposes IFNα treatment together with the antiviral drugs lopinavir and ritonavir (http://www.nhc.gov.cn/yzygj/s7653p/202001/f492c9153ea9437bb587ce2ffcbee1fa.shtml), and many clinical trials with combination treatment involving type I IFNs are currently under way (https://clinicaltrials.gov/ct2/results?cond=COVID&term=ifn&cntry=&state=&city=&dist=). It is possible that IFNλ treatment could also benefit from such a combinatorial approach.

In terms of pharmacological formulation, a pegylated IFNλ version is already available (peginterferon lambda, Eiger Biopharmaceuticals) and others are under development. This formulation has already been used to treat more than 3,000 people in clinical trials for hepatitis B, C, and D (https://clinicaltrials.gov/ct2/results?cond=hepatitis&term=peginterferon+lambda&cntry=&state=&city=&dist=), and, although it has not yet been approved, it demonstrated a favorable safety profile. By comparison to type I IFNs, this approach is several times safer in terms of putative side effects (Muir *et al*, 2014), most likely because IFNλs are not pro‐inflammatory (Galani *et al*, 2017). Peginterferon lambda‐1a is now entering proof‐of‐concept phase II studies in patients with mild disease; the primary outcome is the duration of viral presence (shedding) measured by qRT–PCR (NCT04344600, NCT04343976, NCT04331899).

## Conclusions

As COVID‐19 is killing thousands of people each day, there is an urgent need to identify novel and effective therapeutics. Here, we propose the application of IFNλ to help with the two main clinical problems of the disease—persistent virus presence in the lung and induction of a “cytokine storm”—that affect a small but significant vulnerable population and that lead to pneumonia, ARDS, and ultimately death. Pegylated IFNλs already exist and have a safe profile in humans, other formulations are under development. IFNλ therapy to reduce the viral load and prevent massive tissue damage in the lung of patients is a very promising hypothesis that should be tried and tested in the clinic. Given the lack of an efficient vaccine and the prospect of a second wave of infections, such tests should start rather early.

## Conflict of interest

The authors declare that they have no conflict of interest.
